# Elevated expression of Toll-like receptor 4 and cytokines in both serum and myometrium at term may serve as promising biomarkers for uterine activation preceding labor

**DOI:** 10.3389/fendo.2023.1255925

**Published:** 2023-10-05

**Authors:** Zixi Chen, Jinpeng Li, Wenjuan Xu, Xiaomei Wu, Fenfen Xiang, Xiaoxiao Li, Mengzhe Zhang, Jin Zheng, Xiangdong Kang, Rong Wu

**Affiliations:** ^1^ Department of Laboratory Medicine, Putuo Hospital, Shanghai University of Traditional Chinese Medicine, Shanghai, China; ^2^ Department of Obstetrics and Gynecology, Putuo Hospital, Shanghai University of Traditional Chinese Medicine, Shanghai, China

**Keywords:** uterus activation, TLR4, cytokines, inflammation, MAPK/NF-κB

## Abstract

**Objective:**

Increased inflammation and cytokine levels are considered risk factors and promoters of preterm birth (PTB). However, the regulatory mechanism of pregnancy-related inflammation remains unclear. Toll-like receptor 4 (TLR4) plays a critical role in inflammatory responses in various diseases. Therefore, our study aimed to investigate whether TLR4 is involved in the inflammatory responses during uterine activation for labor, with the goal of identifying potential biomarkers for uterine activation at term.

**Materials and methods:**

We used flow cytometry to detect TLR4 expression on CD14+ maternal blood monocytes in the first, second, and third trimesters. ELISA was employed to measure TLR4 and cytokines levels in the maternal serum of term non-labor (TNL), term labor (TL) women and LPS induced preterm labor and PBS injected controls. TLR4siRNA was transfected into the human myometrial smooth muscle cells (HMSMCs), which were subsequently treated with IL-1β. The mRNA and protein levels of TLR4, uterine contraction-related protein connexin 43 (CX43), oxytocin receptor (OTR), MAPK/NF-κB signaling pathway, and cytokines were analyzed using qRT-PCR, western blotting, and immunohistochemistry.

**Results:**

The study revealed TLR4 expression on CD14+ maternal blood monocytes was higher in the third trimester group compared to the first and second trimester groups (p<0.001). Maternal serum concentrations of TLR4 and cytokines were significantly higher in the TL group than the TNL group (p<0.001). TLR4, OTR, CX43, activated MAPK/NF-κB expression, and cytokines levels were upregulated in TL group, and similarly significantly higher in the LPS-induced preterm group than in the control group. Using the HMSMCs we demonstrated that TLR4siRNA transfection suppressed contractility. Interfering with TLR4 expression reduced the expression of OTR, CX43, cytokines, and MAPK/NF-κB activation. There was a significant positive relationship between TLR4 expression and the inflammatory status in the myometrium. ROC analysis indicated that TLR4 and cytokines may serve as potential biomarkers for predicting uterine activation for labor.

**Conclusion:**

Our data suggest that TLR4 and cytokines can act as stimulators of uterine activation for labor at term. Furthermore, the MAPK/NF-κB pathway appears to be one of the potential signaling pathways mediating TLR4’s regulation of parturition initiation.

## Introduction

Preterm birth (PTB), defined as birth occurring before 37 completed weeks of pregnancy, affects 5-18% of all pregnancies and remains the leading cause of perinatal mortality and morbidity worldwide. PTB can lead to long-term disorders such as cerebral palsy, cognitive impairment, blindness, deafness, and respiratory illnesses (1, 2). Unfortunately, effective methods for predicting or preventing preterm birth are currently lacking, mainly due to our limited understanding of the underlying mechanisms that initiate term labor (TL).

Recent research has highlighted that parturition is an inflammatory event characterized by the infiltration of inflammatory cells into the myometrium and cervix. This is accompanied by increased expression of vascular and leukocyte adhesion molecules, as well as elevated production of chemokines and pro-inflammatory cytokines within the uterus (3). The accumulation of leukocytes in gestational tissues triggers the release of a large quantity of pro-inflammatory cytokines, promoting the expression of uterine contraction-associated proteins (CAPs), which include gap junctions, prostaglandin receptors, and oxytocin receptors. These processes ultimately lead to uterine contractions and labor (4–6). Inflammatory activation is a major driving factor behind PTB, and it is responsible for significant perinatal morbidity and mortality in many countries (7, 8). Moreover, surviving preterm births increases the risk of cardiovascular, gastrointestinal, and respiratory diseases (9–11). Despite the importance of understanding the physiological pathways that govern normal term labor, effective methods for predicting or preventing preterm birth are still lacking.

Toll-like receptors (TLRs) are critical regulators of the inflammatory activation that precedes both preterm delivery and normal term labor (12, 13). Monocytes and neutrophils are the primary sources of cytokines during the early stages of inflammation (14, 15), and TLR4 is expressed in neutrophils, monocytes, and macrophages (16, 17). TLR4 is particularly significant due to its ability to integrate a variety of diverse pro-inflammatory triggers, including endogenous agents released during cell senescence or following injury or infection. It serves as a point of convergence through which various triggers can activate parturition (18–20). TLR4 has been found in chorioamniotic membranes during PTB (21), even in cases where infection is not involved. This suggests that TLR4 may also contribute to the effects of sterile stressors such as stretching or ischemic injury, as tissue damage leads to the release of endogenous TLR4 ligands known as damage-associated molecular patterns (DAMPs) (22). Studies on TLR4 knockout mice have shown that TLR4 is an essential upstream regulator of on-time parturition (23), as its absence can delay delivery. Additionally, TLR4 activation has been observed in the gestational tissues of both term and PTB cases in humans (24). Given that TLR4 activation drives pathological inflammatory reactions and is implicated in the development of various diseases, including Alzheimer’s disease, non-alcoholic fatty liver disease, cryopyrin-associated periodic syndromes, atherosclerosis, type 2 diabetes, and asthma, inhibiting TLR4 activation holds promise as a strategy for developing new treatments for inflammatory diseases (25–28).

The objectives of the present study were to investigate the potential roles of TLR4 and cytokines in the regulation of parturition. First, we observed higher expression levels of IL-6 and TLR4 in the myometrium of pregnant women. TLR4 expression on CD14+ maternal blood monocytes was elevated in the third-trimester pregnancy group compared to the first and second trimester groups. Maternal serum concentrations of TLR4 and cytokines were higher in the TL group than in the term non-labor (TNL) group. TLR4, along with uterine contraction-related proteins (CX43/OTR), activated ERK, P38, NF-κB expression, and proinflammatory cytokines, were upregulated in pregnant women after the onset of labor at term. Using an HMSMCs model, we demonstrated that TLR4 siRNA transfection suppressed contractility. Interfering with TLR4 expression reduced the expression of CX43, OTR, inflammatory cytokines, and activated the MAPK/NF-κB pathway. There was a significant positive relationship between TLR4 expression and the inflammatory status in the myometrium. ROC analysis results indicated that TLR4 and cytokines have the potential to serve as biomarkers for predicting uterine activation for labor.

## Materials and methods

### Collection of maternal peripheral blood, serum and myometrium

This study received approval from the Ethics Committee of Putuo Hospital, affiliated with Shanghai University of Traditional Chinese Medicine, Shanghai, China (No. PTEC-A-2023-14 (S)-1), and informed consent was obtained from the patients. Peripheral blood samples were collected from pregnant women admitted to our hospital between March 2020 and August 2021. Based on their pregnancy status, the participants were divided into two groups: term non-labor (TNL, n = 22) and term in labor (TL, n = 22). Human myometrium samples were obtained from women undergoing elective cesarean section in the following groups: TNL (n = 22) and TL (n = 22). The inclusion criteria for the pregnant women in the study were as follows: aged 25-35 years, single pregnancy, and gestational age between 1 and 40 weeks. Pregnant women with obesity, cardiovascular disease, hypertension, diabetes, or other pregnancy complications were excluded from the study. A total of 2 mL of peripheral blood from each pregnant woman was collected in an EDTA tube and processed within 2 h for flow cytometry analysis. Serum samples were collected in test tubes and stored at -80°C for subsequent ELISA detection. The myometrium tissues were washed with cold saline and stored at -80°C for western blot and RT-PCR analysis. The characteristics of the patients are summarized in **Table 1**.

**Table 1:** Characteristics of study participants in term no labor (TNL), term labor (TL) groups.

	TNL	TL
Age	30.00±4.00	29.00±4.00
Height	162.75±4.13	162.50±7.13
Weight	58.40±14.03	56.80±12.20
Weight Index	21.85±5.52	21.00±5.13
Systolic blood pressure	115.50±110.50	117.00±107.75
Diastolic blood pressure	70.50±11.25	69.00±10.50
Systolic blood pressure/Diastolic blood pressure	1.73±1.53	1.72±1.42
Fasting blood sugar	4.20±0.50	4.20±0.40
Creatinine	46.00±10.25	44.00±10.75
Urea nitrogen	3.80±0.63	3.30±0.85**
ALT	10.50±6.00	9.00±5.25
Placenta weight	590.00±22.50	590.00±20.00
Placental thickness	3.90±0.43	3.75±0.30
Umbilical cord length	60.00±10.00	55.00±15.00
Newborn weight	3445.00±801.25	3340.00±577.25

Data were expressed as mean ± SEM. TNL: n = 22; TL: n = 22**P < 0.01 vs (TNL).

### Animal Experiment and Tissue Collection

Six-to-eight-week-old ICR mice were obtained from Shanghai SLAC Laboratory Animal Co., Ltd and housed under pathogen-free conditions at the Laboratory Animal Centre of Putuo Hospital, Shanghai University of Traditional Chinese Medicine. All animal treatments were carried out using humane procedures. Approval was granted by the animal experiments Ethics Committee at Pu Tuo Hospital, which is affiliated with Shanghai University of Traditional Chinese Medicine (No.0018). Female mice were paired with males, and a vaginal plug was detected on gestation day (GD) 0.5. To establish a preterm labour model, pregnant females were subcutaneously injected with LPS at a concentration of 0.5 mg/kg twice on GD15.5. The time of delivery was documented, and myometrial tissue was gathered upon preterm labor. All samples of myometrial tissue were preserved at −80°C for western blot analysis.

### Cell culture

HMSMCs were isolated from TNL myometrial tissues following the previously described protocol (29). In brief, myometrial tissue pieces were incubated with DMEM supplemented with 1 mg/mL collagenase type II (Invitrogen) and 1 mg/mL deoxyribonuclease I at 37°C with shaking for 30 min, repeated twice. The cell suspension obtained after filtration was centrifuged, and the cell pellet was resuspended in DMEM supplemented with 10% fetal calf serum (FCS), penicillin (100 U/mL), and streptomycin (100 mg/mL). The cells were then seeded into 25 cm (2) flasks and cultured at 37°C in a 5% CO_2_–95% air-humidified atmosphere until reaching confluence, which typically took around 2 weeks. The experiments were conducted using cells at passage 2.

### Flow cytometry analysis

In this study, we used flow cytometry to detect the surface expression of TLR4 on CD14+ maternal blood monocytes in different trimesters of pregnancy. The 1st trimester group consisted of pregnancies before 12 w, the 2nd trimester group included pregnancies between 12 and 27 w, and the 3rd trimester group encompassed pregnancies at or beyond 28 w. To identify monocytes, we employed a gating strategy using forward scatter-A (FSC-A) and side scatter-A (SSC-A) parameters to isolate the monocyte population. Whole blood samples (100 μL) were incubated with 10 μL of the following anti-human antibodies in the dark at room temperature for 15 min: Allophycocyanin (APC)-labeled anti-CD14 (340436; BD Biosciences) and fluorescein isothiocyanate (FITC)-labeled anti-human CD284 (TLR4) (53-9917; eBiosciences). Red blood cells were subsequently lysed using 450 μL of a lysing solution. A total of 10,000 CD14+ monocytes and neutrophils were analyzed for each sample.

### Western blotting analysis

Proteins were extracted from human and mice myometrium using RIPA buffer. The protein concentrations were determined using the BCA Protein Assay Kit (23227, Pierce). A total of 50 μg of protein samples was resolved on a 10% SDS-PAGE gel. Nitrocellulose membranes were then incubated overnight at 4°C with specific primary antibodies: TLR4 (AF7017, Affinity), ERK (BF8004, Affinity), Phospho-ERK (BF8011, Affinity), OTR (AF4667, Affinity), CX-43 (BF8212, Affinity), JNK (AF6318, Affinity), Phospho-JNK (AF3318, Affinity), P65 (AF5006, Affinity), Phospho-P65 (AF2006, Affinity), P38 (8690S, Cell Signaling), and Phospho-P38 (4511S, Cell Signaling), all at a dilution of 1:1000. Subsequently, the nitrocellulose membranes were incubated with secondary fluorescent antibodies at a dilution of 1:5000 for 1 h at room temperature. Band densitometry was analyzed using Quantity One imaging software (Bio-Rad, Hercules, CA, USA).

### Total RNA extraction and quantitative PCR analysis

Total RNA was extracted from tissues and cells using TRIzol (Invitrogen). The cDNA was synthesized from the extracted total RNA using the primeScriptTM RT Master Mix kit (Perfect Real Time, Takara). Specific primers for TLR4, IL-1β, IL-6, TNF-α, IL-8, and IL-10 were designed by Sangon company (Shanghai) and are listed in **Table 1**. Each reaction mixture contained 2.0 µl of cDNA product, 0.2 µmol/L of each primer, 10 µL of SYBR Green PCR Master Mix (Takara), and sterile water was added to a final volume of 20 µL. RT-PCR was performed using the following program: 2 min at 50°C, 10 min at 95°C, followed by 40 cycles of 20 s at 95°C and 1 min at 60°C. The mRNA expression levels of the target genes were normalized to GAPDH mRNA. The 2-ΔΔCt method was employed for the analysis. The human TLR4, IL-1β, IL-6, TNF-α, IL-8, and IL-10 sequences are presented in **Table 1**.

### Analysis of myometrial cell contractility

Collagen matrix contractility measurement is a method used to assess the contractility of cultured myometrial cells (30). The procedure involves the following steps: 1) Rinse the cells, which should be in a good growth state, twice with DPBS, and dilute 0.25% trypsin with DPBS in a 5-fold ratio before digestion. 2) Once the cells become rounded, terminate the digestion and suspend the cells. 3) Transfer the cells into a 15 mL centrifuge tube and centrifuge for 5 min. 4) Remove the supernatant and add 1 mL of Dulbecco’s Modified Eagle Medium (DMEM) to resuspend the cells. 5) Take 20 μL of the cell suspension and add it to a cell counting board. Count the cells in four different areas on the cell counter, and calculate the average. Multiply the average value by 10^4^ to obtain the total number of cells in 1 mL of the cell suspension. 6) Prepare a 12-well plate for collagen contraction, with 1 × 10^5^ cells per well. 7) Prepare the rat tail collagen solution for each well using the following composition: 750 μL DMEM, 250 μL rat tail collagen, and 5 μL NaOH (1 mol/L). 8) Immediately after preparation, resuspend the cells and add them to the 12-well plate, with 1 ml per well. Place the plate in a 37°C, 5% CO_2_ incubator and allow it to stand for 30 min. Remove the plate from the incubator and add 1 mL of complete culture medium (or dissolved medicine) to each well. Use a small pipette tip to draw a circle along the edge of each well, and then gently shake the plate to ensure complete suspension of the gel. Place the plate back into the incubator and record the shrinkage of the gel at 12-h intervals using a digital camera.

### TLR4 RNA interference

The small interfering RNA (siRNA) targeting TLR4 was custom designed and synthesized by GenePharma Corporation (Shanghai, China). A scrambled sequence without any specific known target was used as the control siRNA. The specific sequence for targeting human TLR4 was as follows: sense: 5′-AGACTACTACCTCGATGATAT-3′. The transfection of siRNA was carried out using LipofectamineTM 3000, following the protocol described in a previous study (Chen et al., 2021).

#### Detection of TLR4 and cytokines

Serum levels of TLR4 were quantified using an ELISA kit, while IL-1β, IL-6, IL-8, IL-10, and TNF-α levels were measured using the C2000 electrochemiluminescence immunoassay system (Beijing Hotjing Biotechnology Co., Ltd). All procedures were conducted in accordance with the manufacturers’ instructions.

#### Immunohistochemistry

Paraffin sections (5 μm) were cut and subjected to rehydration. Antigen retrieval was performed using microwave heating in citric acid buffer. Endogenous peroxidase activity was inhibited with 3% H_2_O_2_, and antibody binding was blocked with 10% rabbit serum. The sections were then incubated with the primary antibody targeting TLR4 (AF7017, Affinity). Detection of the antibody was carried out using a biotin-streptavidin peroxidase system (hypersensitive SP kit, Maxin Biotechnology, Fuzhou, China), with diaminobenzidine (Sigma Aldrich, MO) used as the chromogen. Counterstaining was performed using hematoxylin. For negative control, the primary antibody was replaced with IgG.

### Statistical analysis

All data analysis was performed using SPSS 20.0 software. The data are presented as mean ± standard error of the mean (SEM). A one-way Analysis of variance (ANOVA) followed by the Student-Newman-Keuls test was conducted for normally distributed data. In cases where the data did not meet the normal distribution assumption, the Kruskal-Wallis or Mann-Whitney U test was used. Pearson’s correlation analysis was employed to investigate the relationships between TLR4 and cytokine levels. The diagnostic value of the selected parameter in distinguishing between the TNL and TL groups was evaluated using receiver operating characteristic (ROC) curves, and the area under the ROC curve (AUC) was calculated. Cutoff values were determined based on Youden’s index from the ROC curve. A significance level of P < 0.05 was considered statistically significant.

## Results

### Clinical characteristics and laboratory data of the pregnant women

In this study, we analyzed the clinical characteristics and laboratory data of pregnant women in the TNL and TL groups, as shown in **Table 2**. There were no significant differences observed between the two groups in terms of the following variables: maternal age, maternal height, weight, body mass index (BMI) at delivery, systolic pressure, diastolic pressure, systolic/diastolic blood pressure, fasting blood glucose, creatinine, ALT, placental weight, placental thickness, and umbilical cord length (p > 0.05). However, the urea nitrogen levels in the TL group were significantly lower than those in the TNL group (p < 0.001).

**Table 2:** The target primer sequence of human TLR4, IL-1β、IL-6、IL-8、IL-10、TNF-α.

Target	Primer sequence
TLR4	F-CTTATAATTTCGGATGGCAAC
	R-GTGGCTCATATTTAGTACCTG
P65	F-AGTCAGCGCATCCAGACCAAC
	R-CCGCACAGCATTCAGGTCGT
P38	F-CATGCGAAAAGAACCTACAGA
	R-CATATGTTTAAGTAACCGCAGT
ERK	F-CAAGGGCTACACCAAGTCCA
	R-ACTCGGGTCGTAATACTGCT
JNK	F-TCAAGAAGCTAAGCCGACCA
	R-GGCGTCATCATAAAACTCGTTC
IL-1β	F-GATCACTGAACTGCACGCTCC
	R-AGTTATATCCTGGCCGCCTT
IL-6	F-GGTACATCCTCGACGGCATC
	R-ATTTTCACCAGGCAAGTCTCC
IL-8	F-TCAGTGCATAAAGACATACTCCA
	R-TAATTTCTGTGTTGGCGCAGT
IL-10	F-TGCCCCAAGCTGAGAACCAAG
	R-TTCACATGCGCCTTGATGTCT
TNF-α	F-CGAACCCCGAGTGACAAGCC
	R-ATCTCTCAGCTCCACGCCAT

### TLR4 expression on maternal CD14+ monocytes

**Figure 1:**
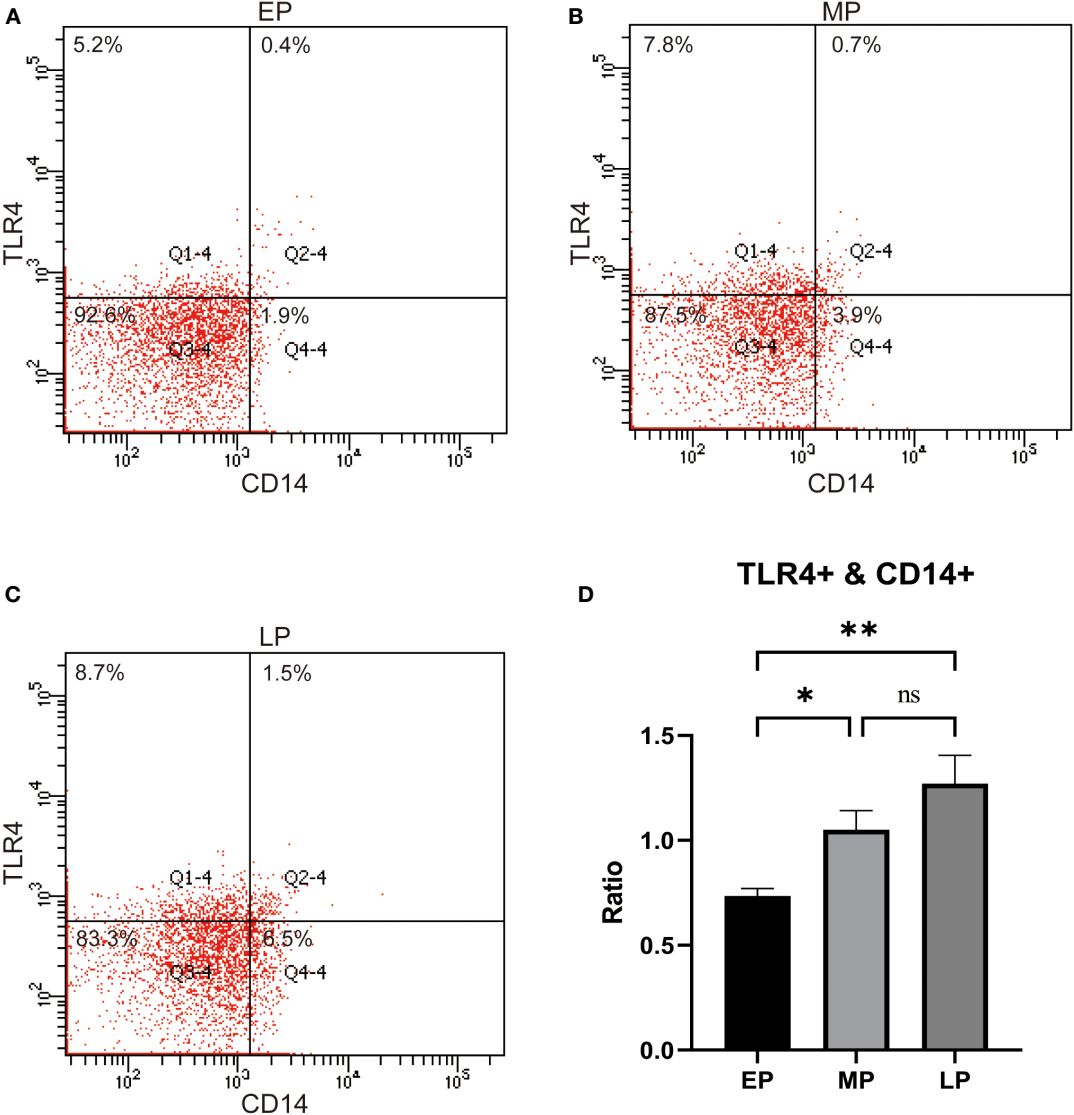
TLR4 expression on CD14+ maternal monocytes in the 1st trimester (Early Pregnancy, EP,<12 w), 2nd trimester (Mid Pregnancy, MP, 12-27 w), and 3rd trimeste (Late Pregnancy, LP, ≥28 w,) women groups **(A–D)**. Compared with the EP and MP groups, TLR4 on CD14+ maternal blood monocytes were upregulated in the LP group. Data were expressed as mean ± SEM. EP: n = 12; MP: n = 12; LP: n = 12. *P < 0.05, **P < 0.01.

Next, we examined the expression of TLR4 in peripheral blood monocytes of pregnant women in the first trimester (EP, < 12 w), second trimester (MP, 12-27 w), and third trimester (LP, >27 w). The results revealed that the levels of TLR4 on CD14+ maternal blood monocytes were significantly higher in the third trimester compared to the first and second trimesters (p < 0.001, as depicted in **Figures 1A–D**).Top of Form

### Upregulation of TLR4 and cytokine expression in human serum following onset of labor

**Figure 2:**
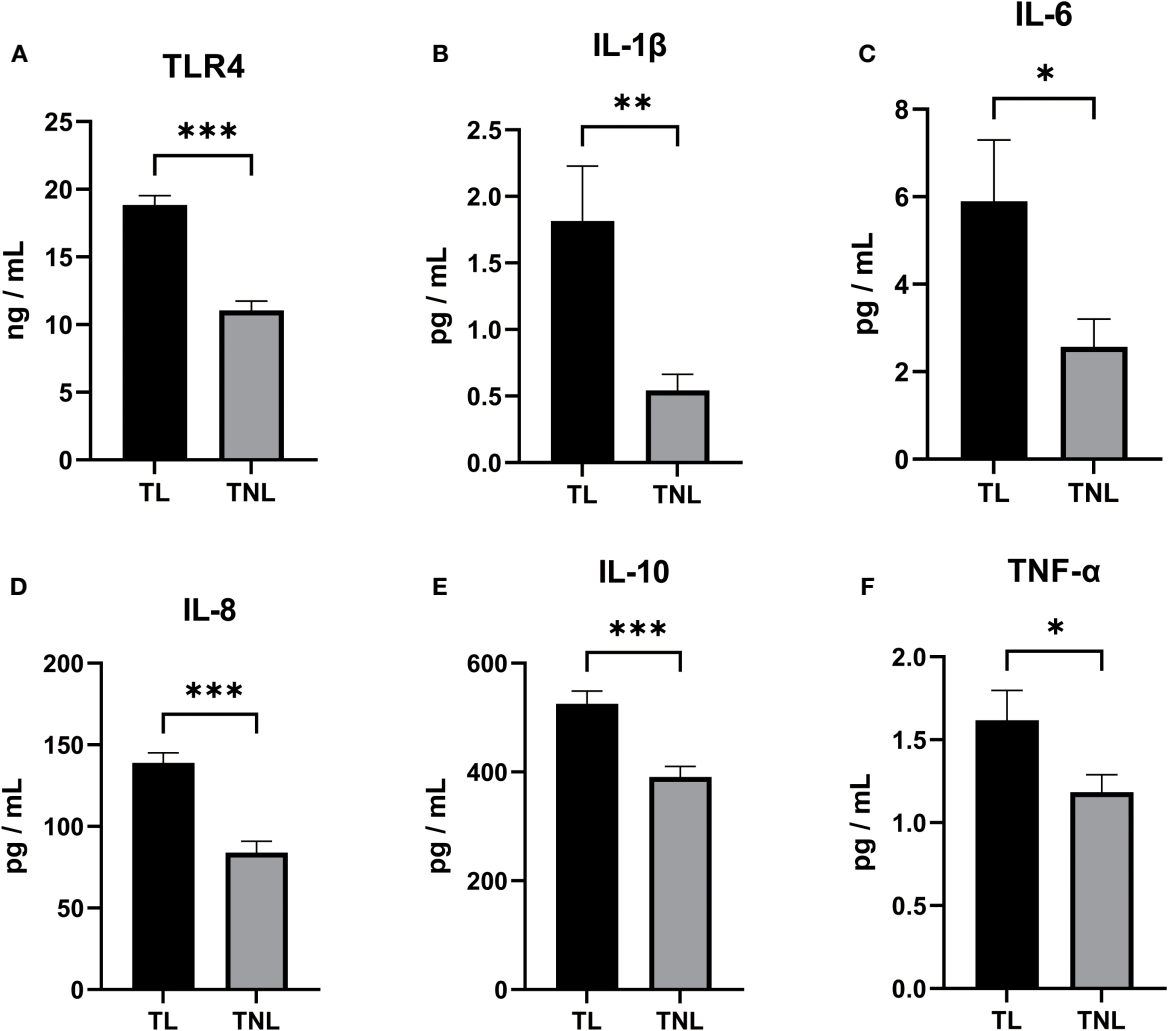
TLR4, Cytokine (IL-1β、IL-6、IL-8、IL-10、TNF-α) concentration in serum of pregnant women before and after the onset of labor at term. **(A–D)** TLR4 concentration **(A)**, IL-1β **(B)** and IL-6 **(C)** and IL-8 **(D)** and TNF-α **(E)** and TNF-α **(F)** concentration in serum of TNL and TL groups. Serum samples were collected from pregnant women with or without labor at term. Data were expressed as mean ± SEM. TNL: n = 22; TL: n = 22. *P < 0.05, **P < 0.01, ***P < 0.001.

Initially, we assessed the expression of TLR4 and proinflammatory cytokines in the serum of patients with TL and TNL. As depicted in **Figure 2A**, the expression of TLR4 in the TL group was significantly higher than in the TNL group (p < 0.001), which is consistent with the findings from sequencing analysis. Furthermore, as shown in **Figures 2B–F**, the levels of proinflammatory cytokines (IL-1β, IL-6, IL-8, IL-10, and TNF-α) were significantly elevated in the serum of the TL group compared to the TNL group (p < 0.05).

### Upregulation of TLR4 and cytokine expression in human myometrium following onset of labor

**Figure 3:**
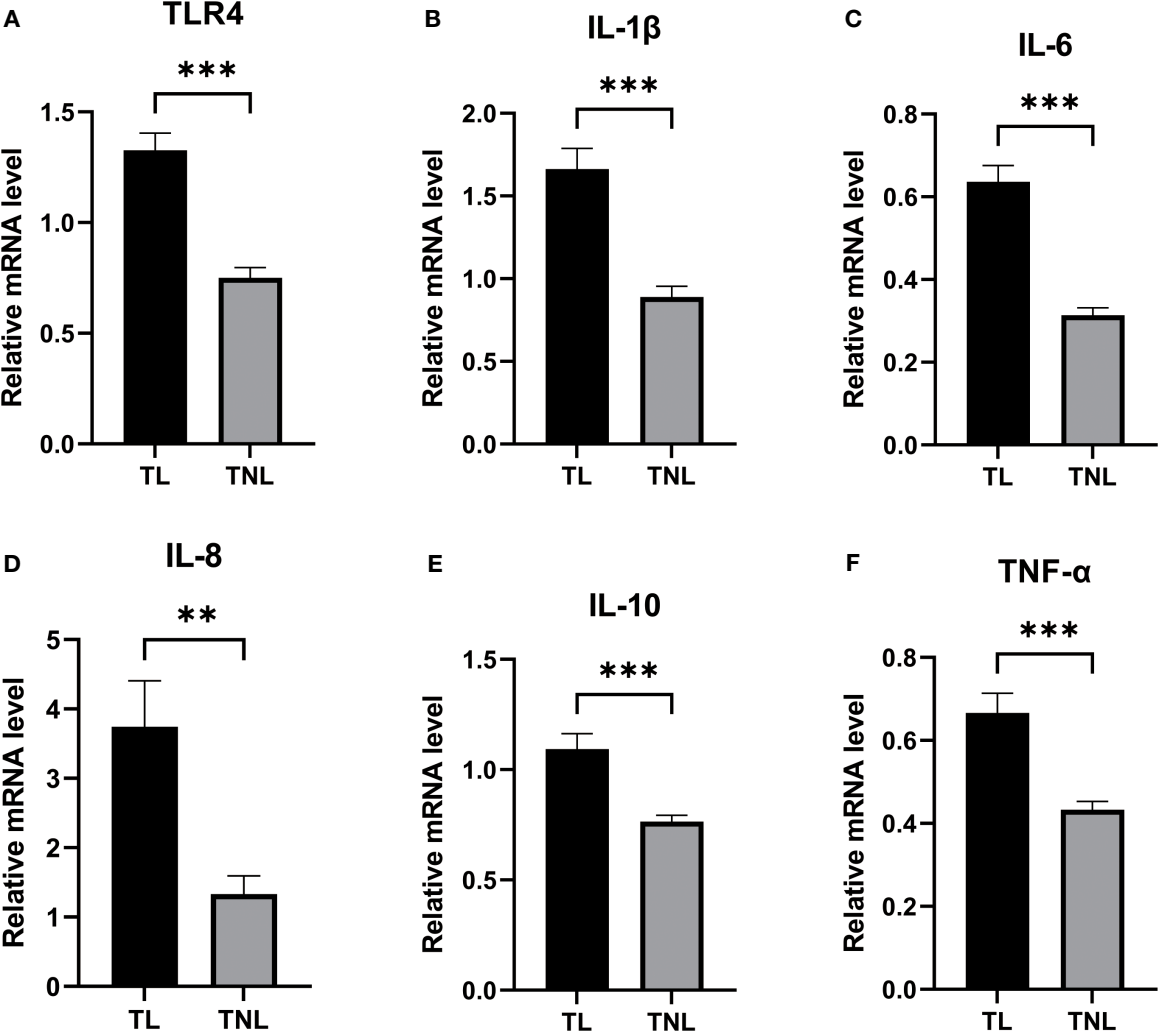
Expression of TLR4 and Cytokine (IL-1β、IL-6、IL-8、IL-10、TNF-α) in human pregnant myomentrium before and after onset of labor. **(A–F)** TLR4 and Cytokine (IL-1β、IL-6、IL-8、IL-10、TNF-α) expression in human myometrium. Myometrial samples were collected from pregnant women before (TNL) and after onset of labor (TL) at term. Real-time PCR was performed to determine mRNA levels of TLR4 and cytokine (IL-1β、IL-6、IL-8、IL-10、TNF-α) expression in myometrium **(A–F)**. Data were expressed as mean ±SEM. TNL: n = 22; TL: n = 22. **P < 0.01, ***P < 0.001.

Given the crucial role of myometrium in the initiation of parturition, we conducted RT-PCR analysis to examine the expression levels of TLR4 and cytokines in both the TL and TNL groups. As depicted in **Figures 3A–F**, the mRNA expression level of TLR4 in the myometrium was significantly higher in the TL group compared to the TNL group (p < 0.05). Additionally, the mRNA levels of proinflammatory cytokines (IL-1β, IL-6, IL-8, IL-10, and TNF-α) were significantly elevated in the TL group compared to the TNL group (p < 0.05).

### LPS-induced preterm labour induces TLR4 and cytokines activation

**Figure 4:**
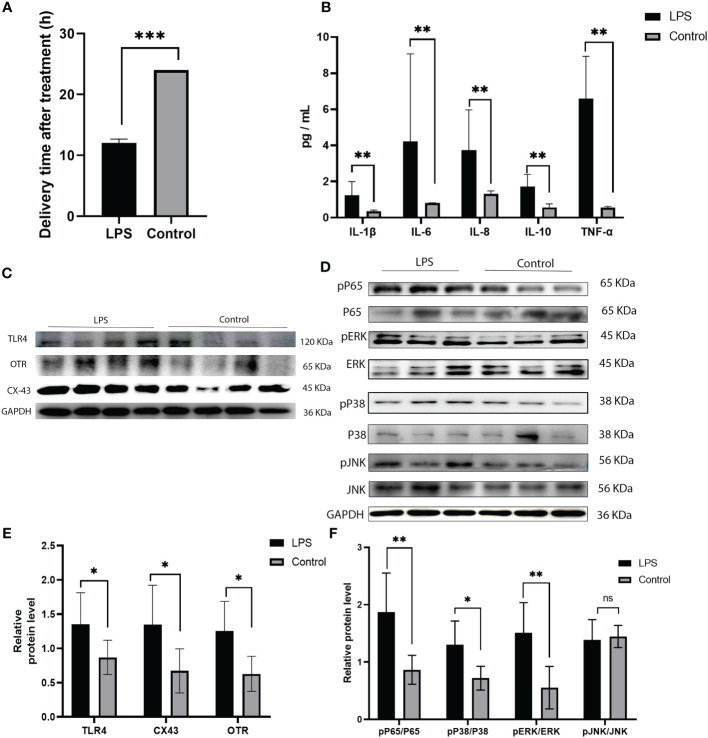
Expression of TLR4, and MAPK/NK-κB activation as well as expression of UAPs (OTR, CX43) in myomentrium of LPS induced preterm labor and PBS treated control group. **(A)** The representative sections of TLR4 expression in LPS induced preterm labor and PBS treated control myomentrium. Myometrium samples were obtained from LPS induced mice preterm labor and PBS treated control. **(A)** Recorded length of labour in mice after LPS and PBS treatments. **(B)** Cytokine (IL-1β、IL-6、IL-8、IL-10、TNF-α) expression in mice myometrium. **(C–F)** The protein level of TLR4, MAPK/NK-κB activation as well as expression of UAPs (OTR, CX43 were determined by Western blotting analysis. Representative bands were listed on top of histogram. Data were expressed as mean ± SEM. (n= 6). *P < 0.05, **P < 0.01, ***P < 0.001; P>0.05, not significant.

In this study, we analysed the protein expression of TLR4 and the levels of cytokines in LPS induced preterm labor. The levels of TLR4 were significantly elevated in LPS-induced preterm labour compared to PBS-injected controls (P < 0.01, as shown in **Figures 4C, E**). The cytokines (IL-1β, IL-6, IL-8, IL-10, and TNF-α) were also noticeably higher in LPS-induced preterm labour than in PBS-injected controls (P < 0.01, as indicated in **Figure 4B**).

### Correlation between TLR4 and inflammatory cytokines in human serum following onset of labor

**Figure 5:**
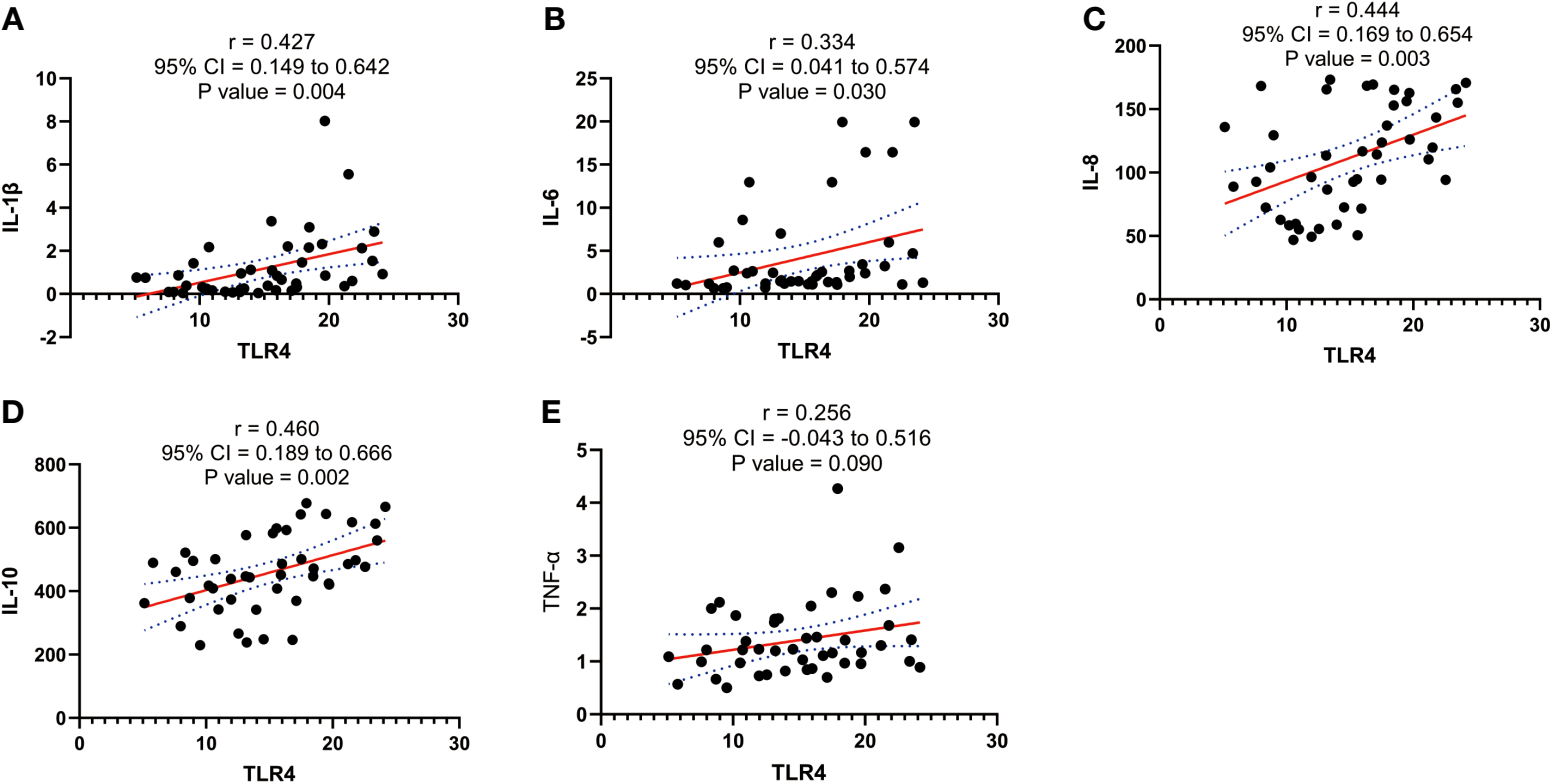
Correlation analysis between TLR4 with cytokine (IL-1β、IL-6、IL-8、IL-10、TNF-α) concentration in serum of pregnant women before and after the onset of labor at term. **(A)** Correlation analysis between TLR4 with IL-1β. **(B)** Correlation analysis of TLR4 with IL-6. **(C)** Correlation analysis between TLR4 with IL-8. **(D)** Correlation analysis of TLR4 with IL-10. **(E)** Correlation analysis between TLR4 with TNF-α. Data were expressed as mean ± SEM. TNL: n = 22; TL: n = 22. P>0.05, not significant.

Our experimental findings demonstrate a significant association between TLR4 and inflammatory cytokines (IL-1β, IL-6, IL-8, IL-10, and TNF-α) in the initiation of uterine delivery. The expression levels of these factors undergo significant changes during human parturition. To further investigate their relationship, we conducted correlation analysis and observed positive correlations between TLR4 and IL-1β, IL-6, IL-8, and IL-10 (p=0.004, p=0.03, p=0.003, and p=0.002, respectively) as shown in **Figures 5A–E**.

### ROC analysis results of TLR4 and inflammatory cytokines as potential biomarkers for monitoring and detecting uterine activation for delivery initiation

**Figure 6:**
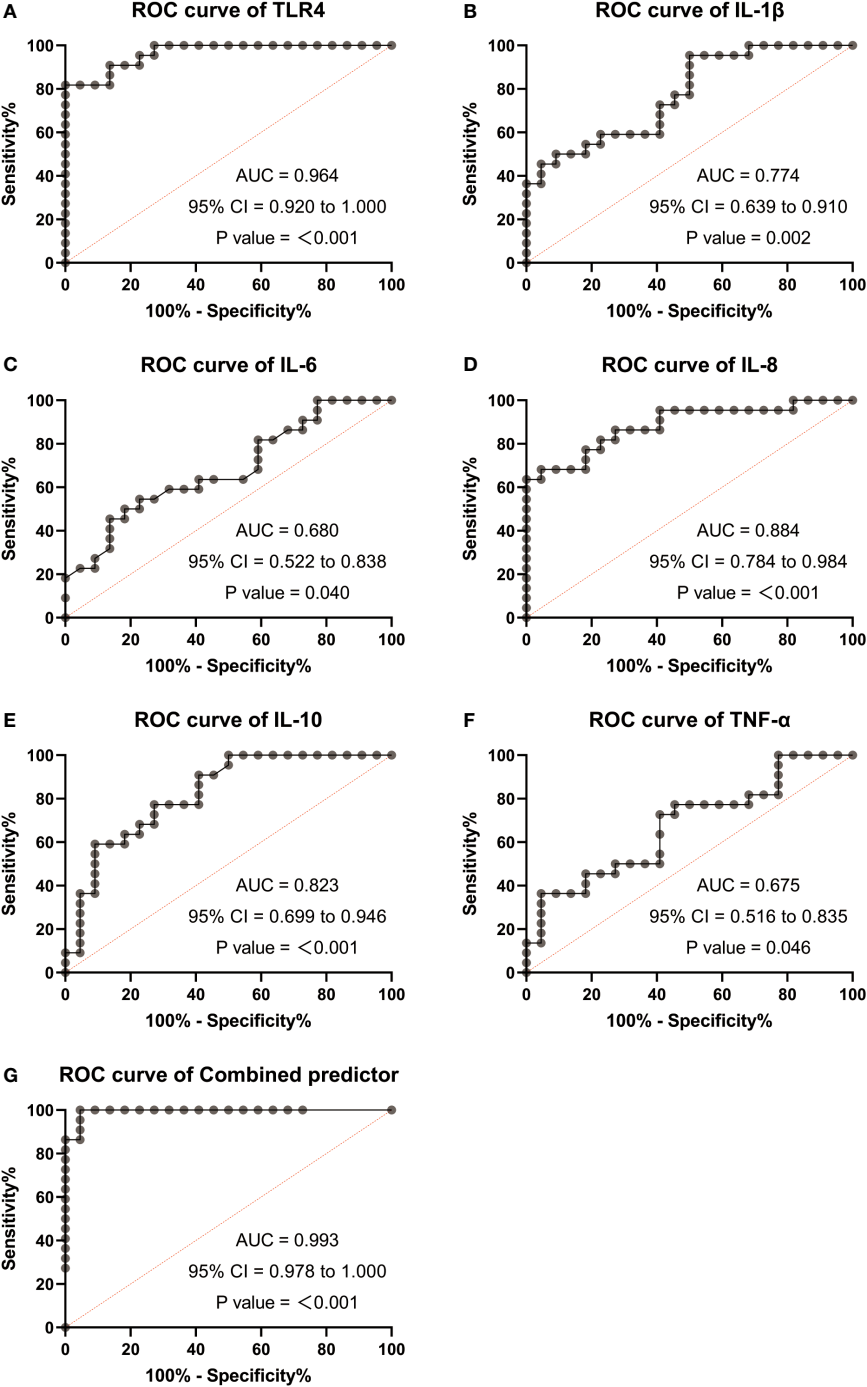
Receiver operating characteristic (ROC) analysis were used to explore the possibility of TLR4 and cytokine (IL-1β、IL-6、IL-8、IL-10、TNF-α) expressing differentially act as potential biomarkers monitoring and detecting uterine activation for delivery initiation. **(A)** ROC curves of TLR4. **(B)** ROC curves of IL-1β. **(C)** ROC curves of IL-6. **(D)** ROC curves of IL-8. **(E)** ROC curves of IL-10. **(F)** ROC curves of TNF-α. **(G)** ROC curves of different factors (TLR4 and IL-8). TNL: n = 22; TL: n = 22. ***, P <0 .001; **, P < 0.01; *, P <0 .05; P>0.05, not significant.

Based on the significant differences observed in TLR4 and inflammatory cytokines (IL-1β, IL-6, IL-8, IL-10, TNF-α) between TL patients, we performed ROC curve analysis to assess their diagnostic value. The AUC for TLR4 in the TL group was 0.964 (0.92-1.0), while for IL-1β, it was 0.774 (0.639-0.91), IL-6 0.68 (0.522-0.838), IL-8 0.884 (0.784-0.984), IL-10 0.823 (0.699-0.946), and TNF-α 0.675 (0.516-0.835). The ROC curve for the combined factors (TLR4, IL-1β, IL-6, IL-8, IL-10, and TNF-α) yielded an AUC of 0.993 (0.978-1.0) (**Figures 6A–G**). These results suggest that TLR4 and inflammatory cytokines have the potential to serve as biomarkers for predicting uterine activation for labor.

### Involvement of TLR4 and MAPK/NF-κB signaling in the activation of parturition in pregnant myometrium

**Figure 7:**
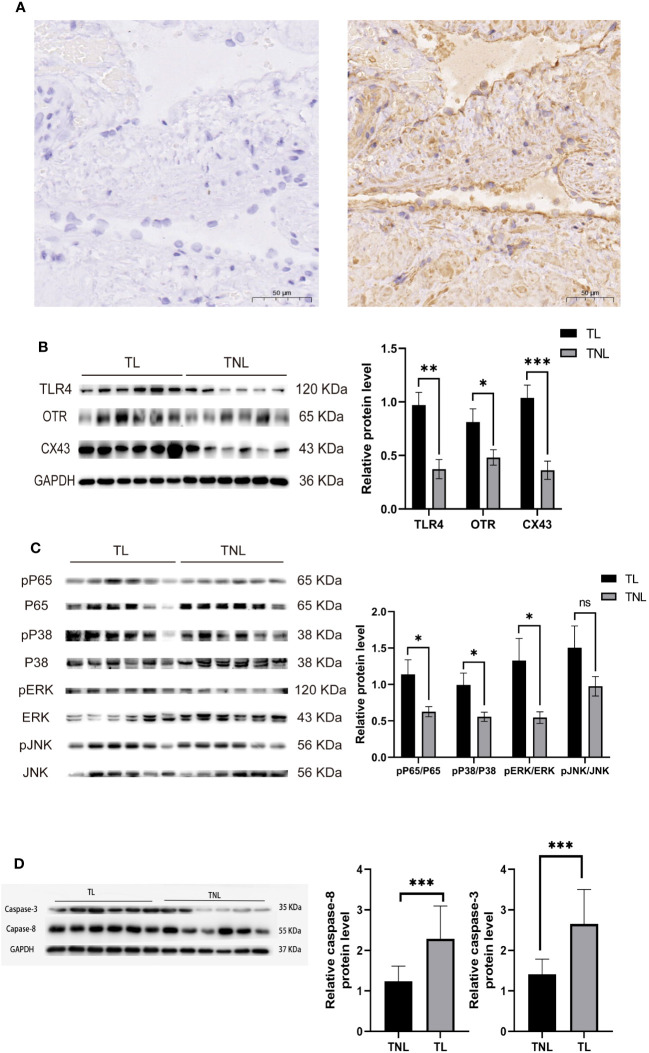
Characterization of TLR4, and MAPK/NK-κB activation as well as expression of UAPs (OTR, CX43) in myomentrium of pregnant women before and after the onset of labor at term. Myomentrium were obtained for determination of TLR4 and MAPK/NF-κB activation and the expression of UAPs. **(A)** The representative sections of TLR4 expression in pregnant women myomentrium at term. Myometrium samples were obtained from healthy pregnant women before and after the onset of labor at term. The tissue sections were used for TLR4 immunocytochemistry analysis. Arrows indicate the positive staining of TLR4. **(B, C)** The protein level of TLR4 and UAPs was determined by Western blotting analysis. Representative bands were placed on the left of corresponding histogram. **(D)** The protein level of MAPK/NK-κB was determined by Western blotting analysis. Representative bands were listed on top of histogram. Data were expressed as mean ± SEM. (n= 12). *P < 0.05, **P < 0.01, ***P < 0.001, ****P < 0.0001; P>0.05, not significant.

Immunohistochemistry was performed to detect the distribution of TLR4 in collected myometrium samples. The immunohistochemical analysis revealed that TLR4 was expressed in the pregnant myometrium and predominantly localized in smooth muscle cells (**Figure 7A**). We further investigated the expression of TLR4, uterine contraction-related protein (CX43/OTR), and key signaling pathways involved in mediating the inflammatory response (NF-κBp65, MAPK - JNK, ERK, and P38) in the human and mice myometrium using western blot analysis. As depicted in **Figures 7B, C**, the protein levels of TLR4, CX43, and OTR were significantly higher in the human myometrium of the TL group compared to the TNL group (p < 0.05), and similarly in the LPS-induced mice preterm labour group than in the control group (**Figures 4D, F**) (p < 0.05). Similarly, the protein levels of NF-κBp65, ERK, and P38 signaling pathways were significantly elevated in the human myometrium of the TL group compared to the TNL group (p < 0.05), and significantly higher in the LPS-induced mice preterm labour group than in the control group (**Figures 2B, C**), (p < 0.05). However, no significant differences were observed in JNK mRNA levels (p > 0.05) (**Figures 7D, E**, **4D, F**).

### TLR4 promotes contractility, upregulates inflammatory cytokine expression, uterine activation proteins and activates NF-κB/MAPK in cultured HMSMC

**Figure 8:**
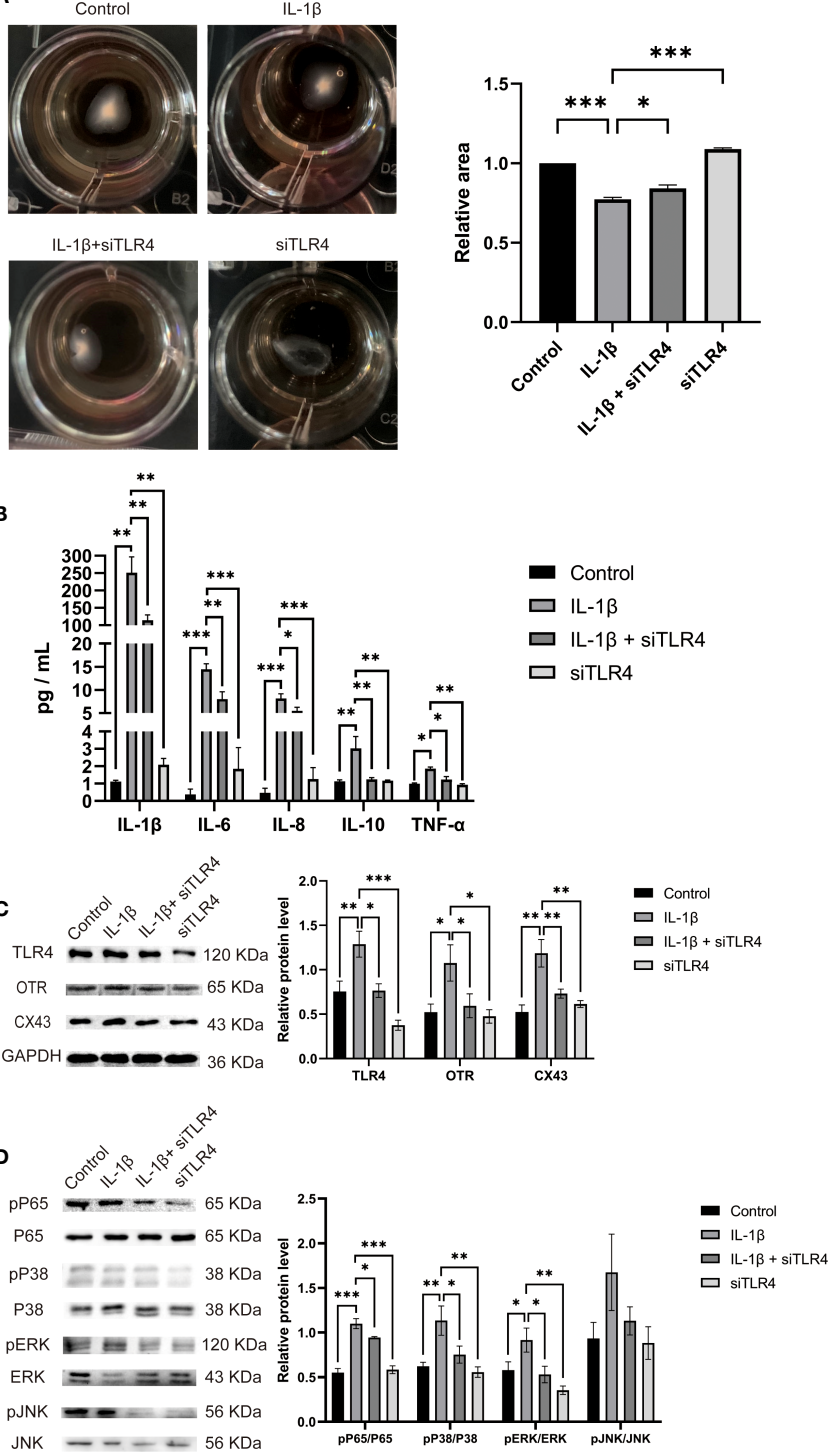
TLR4 promotes contractility and UAPs expression and MAPK/NF-κB activation of cultured HMSMCs. **(A–C)**, TLR4 knockdown on contractility of HMSMCs. Cultured HMSMCs were pretreated with IL-1β, transfected with TLR4 siRNA. **(A)** The contractility was determined by Collagen Matrix Contractility Assay. **(B)** TLR4 promotes mRNA level of cytokine (IL-1β、IL-6、IL-8、IL-10、TNF-α) in cultured HMSMCs was determined by RT-PCR analysis. **(C)** The protein levels of TLR4、COX-2、CX43 were determined by western blotting. **(D)** The protein levels of MAPK/NF-κB were determined by western blotting. Representative bands were listed on top of histogram. Data were expressed as mean ± SEM (n = 4 independent cultures). *P < 0.05, **P < 0.01, ***P < 0.001, P>0.05, not significant.

The transition of the myometrium from a resting state to a contractile state is a crucial step in the initiation of labor. This transition is closely associated with myometrial cell contractility. Therefore, we investigated the role of TLR4 in regulating myometrial contractility and facilitating labor initiation using cultured HMSMCs as a model. As expected, cultured HMSMCs exhibited spontaneous contractions, evident by the reduced areas of collagen lattices at 24 h (**Figure 8A**). To confirm the involvement of TLR4 in controlling HMSMCs’ contractility, we examined the effects of TLR4 siRNA knockdown on contraction. Consistent with our expectations, transfection of HMSMCs with TLR4 siRNA resulted in a significant decrease in TLR4 expression (**Figure 8C**). The IL-1β+siTLR4-treated cells exhibited reduced contractility compared to the cells treated with IL-1β alone, as indicated by an increased area of collagen lattices (**Figure 8A**). Considering our previous findings regarding the association between myometrial contractility and inflammatory cytokines, we also assessed the expression levels of inflammatory cytokines (IL-1β, IL-6, IL-8, IL-10, and TNF-α) in HMSMCs upon TLR4 siRNA treatment using ELISA. As shown in **Figure 8B**, treatment of HMSMCs with IL-1β+TLR4 siRNA resulted in a decreased expression of inflammatory cytokines compared to IL-1β treatment alone. TLR4 siRNA also decreased the expression levels of these cytokines compared to IL-1β treatment. Furthermore, we investigated the expression of uterine activation proteins (UAPs), such as COX-2 and CX43, as well as the activation levels of MAPK (p-p38, p-PERK) and NF-κB p-p65 in HMSMCs upon TLR4 siRNA treatment. As depicted in **Figures 8C, D**, treatment of HMSMCs with IL-1β+TLR4 siRNA led to reduced expression of COX-2 and CX43, as well as decreased activation of MAPK (p-p38, p-PERK) and NF-κB p-p65 compared to IL-1β treatment alone. TLR4 siRNA also decreased the expression of COX-2 and CX43, as well as the activation of MAPK (p-p38, p-PERK) and NF-κB p-p65 compared to IL-1β treatment. Based on our findings, it can be inferred that TLR4 and proinflammatory cytokines play a stimulating role in uterine activation for labor at term. Moreover, the MAPK/NF-κB signaling pathway appears to be one of the potential pathways mediating the regulatory effects of TLR4 on the initiation of parturition.

## Discussion

Preterm labor is a pressing public health issue that deserves attention as it is a leading cause of perinatal mortality and disability (29). The process of initiating delivery in preterm labor is similar to that of term labor, albeit occurring earlier than expected (30). However, the precise mechanisms underlying the initiation of labor are not yet fully understood (31, 32). Recent studies suggest that the initiation of labor involves an inflammatory response (31, 32).

There is an increased inflammatory activity during the third trimester of pregnancy, which is characterized by elevated expression of various cytokines including IL-1β, IL-6, TNF-α and a range of chemokines like CCL2 and IL-10, in the uterus, cervix, placenta, and other reproductive tissues (33, 34). Cytokines and chemokines expression leads to the accumulation of leukocytes, mainly macrophages and neutrophils, in the placenta, fetal membranes, decidua, myometrium, and cervix (34). This leads to cervical dilation, rupture of membranes and uterine contractions (33, 35). Infections that initiate the inflammatory pathway are responsible for 25-40% of premature human deliveries (36, 37). Intrauterine infections can occur either systemically or by the ascent of bacteria from the female genital tract into the uterus. Progesterone has a key function in maintaining the inactive state of the uterus during pregnancy. Throughout human pregnancy, progesterone levels maintain a constant high level. Nonetheless, there is a ‘functional’ withdrawal of progesterone in women towards the end of pregnancy (38). New studies indicate that progesterone helps to maintain a state of inactivity in the uterus, hence reducing the production of inflammatory mediators such as cytokines and prostaglandins (39). Study reported that administering RU38486 to pregnant mice may increase the levels of pro-inflammatory cytokines and cause leukocyte infiltration in the myometrium, suggesting a relationship between RU38486-induced PTB and inflammation (40). However, the specific mechanisms behind modulating the inflammatory response in the uterus remain unclear.

Toll-like receptors (TLRs) are crucial protein molecules involved in the body’s nonspecific immune response, acting as a bridge between terms innate and adaptive immunity (41). Current research indicates that TLR4 can promote preterm birth by upregulating the expression of pro-inflammatory factors and chemokines (42). Studies have shown similarities in the changes of white blood cells between infection-induced preterm labor and non-infected full-term labor, which can be attributed to the direct or indirect effects of TLR4 (43). During the late stages of pregnancy, the body releases various endogenous factors that activate TLR4, which plays a key role in driving uterine activation through an inflammatory cascade reaction (22, 44, 45). CD14 is known for its protective effect against bacterial invasion in monocytes and neutrophils (45, 46). Monocytes and neutrophils can contribute significantly to immune defense through TLR4 (45, 47). In this study, we collected ten human uterine muscle tissue samples from clinical subjects before and after full-term labor for RNA seq sequencing. Enrichment analysis was conducted on downstream pathways, revealing significant differences between the two groups in cytokine-cytokine receptor interaction, chemokine signaling pathway, and Toll-like receptor signaling pathway. Given the association between delivery initiation and inflammation, we focused on differentially expressed genes associated with inflammation that were commonly found in the three most significantly enriched pathways: TLR4 and cytokines.

We then examined the expression of TLR4 and cytokines in human peripheral blood and serum. The results revealed that the expression levels of TLR4 in CD14+ marked monocytes gradually increased as pregnancy progressed. Additionally, TLR4 expression in the serum of the TL group was significantly higher than that in the TNL group, and similarly in the LPS-induced mice preterm labour group than in the control group, demonstrating statistical significance. Furthermore, we investigated the protein expression of TLR4 in the myometrium using immunohistochemistry, western blot, and RT-PCR. The findings demonstrated that TLR4 is expressed in the uterus with upregulation toward term. To assess the role of TLR4 in regulating myometrial contractility and facilitating labor initiation, we evaluated the effect of TLR4 siRNA on the contractile ability of uterine smooth muscle cells. As anticipated, transfection of HMSMCs with TLR4 siRNA led to a significant decrease in TLR4 expression. The HMSMCs treated with IL-1β+siTLR4 exhibited reduced contractility, as evidenced by an increased area of collagen lattices. These findings suggest the involvement of TLR4 in uterine activation for labor. Consequently, our study contributes to the understanding of TLR4 functions in reproduction.

Inflammatory activation plays a central and primary role in the processes of parturition, characterized by the accelerated influx of inflammatory leukocytes and synthesis and release of cytokines in the serum and gestational tissues (31). In our study, we assessed the expression levels of cytokines in the serum of TNL and TL pregnant women. The results demonstrated a significant increase in the expression of proinflammatory cytokines (IL-1β, IL-6, IL-8, IL-10, TNF-α) in the serum of the TL group compared to the TNL group, and similarly in the LPS-induced mice preterm labour group than in the control group. These findings suggest that the inflammatory response plays a significant role in the process of human parturition.

Numerous studies have demonstrated the regulatory role of TLR4 in the activation of proinflammatory cytokines and leukocytes during infection-induced preterm labor (44). In our study, we investigated the correlation between TLR4 expression and proinflammatory cytokines (IL-1β, IL-6, IL-8, IL-10, TNF-α) and found a positive correlation between TLR4 and IL-1β, IL-6, IL-8, and IL-10. To explore the potential of TLR4 and proinflammatory cytokines as molecular markers for monitoring and predicting uterine activation, we conducted ROC curve analysis. Interestingly, the AUC values for TLR4, IL-1β, IL-6, IL-8, IL-10, and TNF-α in the blood of pregnant women were 0.964, 0.774, 0.68, 0.884, 0.823, and 0.675, respectively. The combined AUC of TLR4 and IL-8 was 0.993, suggesting their efficacy as biomarkers for monitoring and predicting uterine activation for labor. Furthermore, we examined the expression of inflammatory cytokines (IL-1β, IL-6, IL-8, IL-10, TNF-α) in HMSMCs upon TLR4 siRNA treatment using ELISA. The results showed that treatment of HMSMCs with IL-1β+TLR4 siRNA reduced the expression levels of inflammatory cytokines compared to IL-1β treatment alone. TLR4 siRNA also decreased the expression levels of inflammatory cytokines compared to IL-1β treatment alone. These findings indicate that TLR4 and proinflammatory cytokines may play a crucial role in the early prediction and monitoring of uterine activation for labor. To the best of our knowledge, no previous study has investigated the combined role of TLR4 and inflammatory factors in human parturition.

Previous research has confirmed that TLR4 can regulate the activation of MAPK and NF-κB, which mediate the inflammatory response (48). The MAPK and NF-κB pathways can modulate the secretion of various inflammatory factors, including IL-1β and TNF-α. This process can stimulate the release of arachidonic acid and activate phospholipid metabolism, leading to the secretion of prostaglandins by uterine muscle cells. Consequently, there is an increase in calcium influx into uterine muscle cells, enhancing uterine contraction and potentially resulting in premature delivery. In our study, we analyzed the protein levels of NF-κBp65, JNK, ERK, and P38 in the human and LPS induced mice myometrium. The results showed that NF-κBp65, ERK, and P38 protein levels were significantly higher in the myometrium of the TL group compared to the TNL group, and similarly in the LPS-induced mice preterm labour group than in the control group. However, no statistical differences were observed in JNK protein levels. Furthermore, we examined the expression of UAPs and the MAPK/NF-κB pathway in HMSMCs upon TLR4 siRNA treatment. The results showed that treatment of HMSMCs with IL-1β+TLR4 siRNA reduced COX2 and CX43 expression, as well as the levels of MAPK (p-p38, p-ERK) and NF-κB p-p65, compared to IL-1β treatment alone. These findings suggest that the MAPK/NF-κB pathway may be one of the potential signaling pathways mediating TLR4 regulation of parturition initiation.

## Conclusion

In conclusion, this study has provided evidence that elevated levels of TLR4 and inflammatory cytokines serve as significant biomarkers for uterine activation leading to labor at term. Furthermore, the involvement of the MAPK/NF-κB signaling pathway has been identified as a partial mediator of the effects exerted by TLR4 and proinflammatory cytokines, highlighting its crucial role in regulating uterine activation for labor.

## Data availability statement

The data presented in this article are not publicly available due to legal and privacy restrictions, specifically concerning patient confidentiality and participant privacy. Requests to access data should be directed to the corresponding author.

## Ethics statement

The studies involving humans were approved by Ethics Committee of Putuo Hospital, affiliated with Shanghai University of Traditional Chinese Medicine, Shanghai, China (No. PTEC-A-2023-14 (S)-1). The studies were conducted in accordance with the local legislation and institutional requirements. The participants provided their written informed consent to participate in this study. The animal studies were approved by animal experiments Ethics Committee at Pu Tuo Hospital, which is affiliated with Shanghai University of Traditional Chinese Medicine (No.DWEC-A-202306002). The studies were conducted in accordance with the local legislation and institutional requirements. Written informed consent was obtained from the owners for the participation of their animals in this study.

## Author contributions

ZC: Writing – original draft, Project administration, Formal Analysis, Funding acquisition. JL: Formal Analysis, Software. WX: Writing – review & editing. XW: Writing – review & editing. FX: Writing – review & editing. XL: Writing – review & editing. MZ: Writing – review & editing. JZ: Writing – review & editing. XK: Writing – review & editing. RW: Writing – review & editing.
